# Dose–response toxicity and locomotor effects of *Mucuna pruriens*-based phytotherapeutic combinations in pesticide-exposed *Drosophila melanogaster*

**DOI:** 10.3389/ftox.2026.1930770

**Published:** 2026-09-09

**Authors:** Raphael Mwezi, Maulilio Kipanyula, John – Mary Vianney, Flora Stephano

**Affiliations:** 1 Directorate of Pesticides Management, Tanzania Plant Health and Pesticide Authority (TPHPA), Arusha, Tanzania; 2 Department of Global Health and Biomedical Sciences, Nelson Mandela African Institution of Science and Technology, Arusha, Tanzania; 3 Department of Zoology and Wildlife Conservation, College of Natural and Applied Sciences, University of Dar es Salaam (UDSM), Dar es Salaam, Tanzania

**Keywords:** dose–response toxicity, *Drosophila melanogaster*, glyphosate, locomotor impairment, *Mucuna pruriens*, negative geotaxis, paraquat, phytotherapy

## Abstract

**Background:**

*Mucuna pruriens* contains L-3,4-dihydroxyphenylalanine (L-DOPA) and other bioactive constituents with potential relevance to motor dysfunction; however, the safety of crude extracts and the behavioural effects of multi-botanical formulations remain insufficiently characterized. This study evaluated the concentration-dependent toxicity of crude *M. pruriens* seed extract and its locomotor effects, alone and in combination with *Cucurbita pepo* and *Arachis hypogaea*, in pesticide-exposed adult male *Drosophila melanogaster*.

**Methods:**

For toxicity assessment, flies were exposed for 7 days to diets containing 0.1%–2.0% (w/v) *M. pruriens*. Each treatment comprised nine vials containing 20 flies per vial, giving 180 flies per treatment. Mortality, survival, hazard ratios, no-observed-effect level (NOEL), lowest-observed-effect level (LOEL), and model-derived median lethal concentration (LC_50_) were determined. Locomotor impairment was induced using paraquat at 10 or 20 mM or glyphosate at 0.5%, 1.0%, or 2.0% (w/v). Negative geotaxis was evaluated in three independent experimental runs, with four vials per treatment per run and 10 flies per vial, giving 12 vial-level experimental units and 120 flies per treatment. Vial-level area under the climbing-response curve (AUC) was used as the primary locomotor endpoint.

**Results:**

Seven-day mortality increased from 13.9% in negative control to 45.6% at 2.0% *M. pruriens*. The operational NOEL and LOEL were 0.1% and 0.5%, respectively. Logistic modelling estimated an LC_50_ of 2.06% (95% CI, approximately 1.71%–2.41%); this estimate was model-dependent and extrapolated because mortality remained below 50%. Paraquat and glyphosate significantly reduced vial-level locomotor AUC relative to the negative control. Treatment with 0.1% *M. pruriens*, alone or in combination with *C. pepo* and/or *A. hypogaea*, produced partial locomotor improvement relative to pesticide-only baselines, but none of the treatments restored performance to the negative-control level.

**Conclusion:**

Crude *M. pruriens* seed extract exhibited concentration-dependent toxicity, while the 0.1% concentration was associated with partial locomotor improvement in pesticide-exposed *D. melanogaster*. The findings provide preliminary toxicological and behavioural evidence but do not establish direct neuronal protection or a molecular neuroprotective mechanism. Further studies should include appropriate vehicle and solvent controls, higher concentrations to improve LC_50_ estimation, fixed-total-dose combination formulations, phytochemical standardization, and mechanistic assessment of oxidative stress, mitochondrial integrity, and dopaminergic neurons.

## 1 Introduction

Parkinson’s disease (PD) is a progressive neurodegenerative disorder characterized primarily by degeneration of dopaminergic neurons in the substantia nigra and the development of motor manifestations including bradykinesia, rigidity, resting tremor, and postural instability (Bloem B. R., 2021; Kalia and Lang, 2015; Poewe et al., 2017). Although ageing and genetic susceptibility are major determinants of PD risk, environmental exposures are increasingly recognized as potentially modifiable contributors to disease development (Brown et al., 2024; Fleming, 2017; Jacobs et al., 2020). Epidemiological and experimental evidence has associated exposure to several pesticides with parkinsonian outcomes, although the strength and consistency of these associations vary according to pesticide class, population, exposure route, duration, and exposure assessment methods (Dorsey, 2023; Polito et al., 2016; Sarkar, 2021). Paraquat is widely used in experimental models of Parkinsonism because it can induce oxidative stress, mitochondrial dysfunction, dopaminergic disturbance, and locomotor impairment (Langston, 2017; Moretto and Colosio, 2013). Recent *Drosophila* studies continue to demonstrate paraquat-associated reductions in climbing performance and survival together with oxidative-stress-related effects (Quintero-Espinosa et al., 2024). Glyphosate has also been associated experimentally with oxidative imbalance, mitochondrial disturbance, neuroinflammatory responses, and behavioural alterations; however, evidence linking glyphosate specifically to human PD remains less established than that for paraquat (Cattani et al., 2014; Costas-Ferreira et al., 2022; Dogan et al., 2025). Therefore, the pesticide exposures used in the present study are considered experimental models of pesticide-associated locomotor dysfunction rather than direct representations of human PD causation.

*Drosophila melanogaster* provides a useful experimental system for investigating pesticide-associated locomotor dysfunction because several pathways relevant to PD, including dopamine metabolism, mitochondrial homeostasis, oxidative-stress responses, proteostasis, and neuronal survival, are evolutionarily conserved (Stephano, 2013). Its relatively short life cycle, well-characterized nervous system, genetic tractability, and reproducible behavioural assays further support its use in neurodegeneration research (Cesur et al., 2025; Madabattula et al., 2015; Spierer et al., 2021). Negative geotaxis provides a practical quantitative measure of locomotor performance and has been widely used to characterize motor impairment in toxin-induced *Drosophila* models (Cao, 2017). Importantly, pesticide concentrations used in experimental *Drosophila* models are selected to produce measurable biological effects under controlled laboratory conditions. They should not be interpreted as direct equivalents of occupational, dietary, residential, or environmental human exposure levels. Differences in exposure route, toxicokinetics, metabolism, body size, and duration prevent direct conversion of experimental fly concentrations into human exposure doses. Accordingly, the paraquat and glyphosate concentrations used in the present study were employed as model-induction concentrations to generate reproducible locomotor impairment while maintaining sufficient survival for behavioural assessment.

*Mucuna pruriens* has attracted considerable interest because its seeds are a natural source of L-3,4-dihydroxyphenylalanine (L-DOPA), the metabolic precursor of dopamine, and also contain flavonoids, phenolic compounds, alkaloids, and other bioactive constituents (Gurushankar, 2024; Poddighe et al., 2014; Yadav and R., 2020). Experimental and clinical studies have investigated *Mucuna pruriens* as a potential source of compounds relevant to motor dysfunction and parkinsonian symptoms (Cilia et al., 2017; Hammoud et al., 2025; Poddighe et al., 2014). Nevertheless, crude botanical preparations may also produce concentration-dependent adverse effects, and their phytochemical composition may vary according to plant origin, extraction method, processing, and formulation. Toxicological characterization is therefore necessary before potential behavioural benefits of crude *M. pruriens* extracts are interpreted (Akindele and Busayo, 2011). Other plant materials may contribute bioactive compounds of potential relevance to locomotor function. *Cucurbita pepo* seeds and *Arachis hypogaea* shells contain phenolic and other phytochemical constituents with reported antioxidant properties (Montesano et al., 2018; Peng et al., 2021; Toomer, 2018). These constituents may theoretically complement those present in *M. pruriens*; however, claims of additive or synergistic activity require dose-matched formulations, evaluation of the individual extracts, and formal interaction analyses. In the absence of such a design, numerical improvements observed with multi-botanical formulations should be interpreted cautiously.

The present study therefore integrated a seven-day concentration–response toxicity assessment of crude *M. pruriens* seed extract with behavioural evaluation of *M. pruriens*, alone and in combination with *C. pepo* and *A. hypogaea*, in paraquat- and glyphosate-exposed *Drosophila melanogaster*. Specifically, the study aimed to characterize mortality and survival profiles, identify operational no-observed-effect and lowest-observed-effect levels, estimate a model-derived LC_50_, quantify pesticide-associated locomotor impairment, and determine the extent of treatment-associated locomotor improvement. Because biochemical, molecular, catecholaminergic, mitochondrial, and dopaminergic-neuron endpoints were not measured, the study was designed to provide toxicological and behavioural evidence rather than direct confirmation of neuronal protection or a specific mechanism of action.

## 2 Materials and methods

### 2.1 Study design

A controlled laboratory study was conducted in two complementary components. The first component characterized the concentration-dependent toxicity of crude *Mucuna pruriens* seed extract using seven-day mortality and survival endpoints. The second component evaluated pesticide-associated locomotor impairment and subsequent changes in locomotor performance following treatment with *M. pruriens* alone or in combination with *C. pepo* and *A. hypogaea*. All experiments were conducted using age-matched adult male *Drosophila melanogaster* maintained under standardized laboratory conditions. Treatment allocation was randomized and climbing-assay observations were conducted with treatment identity masked where practicable.

### 2.2 Chemicals, vehicle, and pesticide concentration units

Analytical-grade methanol was used exclusively for extraction of the plant materials. Paraquat dichloride and glyphosate isopropylamine salt, each reported as 98% pure, were obtained from Sigma-Aldrich, United States of America. Paraquat solutions were prepared on a molar basis and expressed as 10 and 20 mM in the locomotor experiments, while preliminary mortality screening additionally included 30 and 40 mM. Glyphosate solutions were prepared on a weight-per-volume basis at 0.5%, 1.0%, and 2.0% (w/v), corresponding to GRA, GRB, and GRC, respectively. Carboxymethyl cellulose (CMC) at 0.5% (w/v) was used as the vehicle for both pesticide preparations and botanical-extract formulations before incorporation into the fly diet (Swamy et al., 2019). The use of different concentration units for paraquat and glyphosate reflected the preparation conventions applied to the two pesticides and did not imply dose equivalence. Paraquat was expressed on a molar basis, whereas glyphosate was expressed as percentage weight per volume. The pesticide concentrations were experimental model-induction doses and were not intended to represent occupational, dietary, residential, or environmental human exposure levels.

### 2.3 Collection and authentication of plant materials

Seeds of *Mucuna pruriens* (L.) DC. and *C. pepo* L., together with shells of *A. hypogaea* L., were collected from the Morogoro Region, Tanzania. Plant materials were authenticated by a qualified botanist in the Department of Botany, University of Dar es Salaam (UDSM), Tanzania. Voucher specimens were deposited in the departmental herbarium and assigned accession numbers RJW 01, RJW 02, and RJW 03 for *M. pruriens*, *C. pepo*, and *A. hypogaea*, respectively. The authenticated materials were cleaned, rinsed with distilled water, shade-dried at room temperature to constant weight, and separately milled into fine powders using a laboratory grinder. The powdered materials were stored in airtight amber containers at 4 °C until extraction.

### 2.4 Preparation of plant extracts

For each plant material, 100 g of powdered material was extracted with 1,000 mL of analytical-grade methanol for 8 h using a Soxhlet apparatus (Singh and Kumar, 2023). Methanol was removed under reduced pressure at approximately 40 °C using a rotary evaporator, after which the extracts were dried to constant weight and stored in airtight amber containers at 4 °C. Extraction yields were 12.6% for *M. pruriens*, 9.4% for *C. pepo*, and 7.8% for *A. hypogaea*. Immediately before administration, the dried extracts were separately suspended in 0.5% (w/v) CMC prepared in distilled water and incorporated into the fly diet (Swamy et al., 2019). The same concentration of CMC was used as the vehicle for preparation of the pesticide-containing diets. Methanol was used only during plant extraction and was intended to be removed completely during rotary evaporation and subsequent drying. However, residual methanol was not analytically quantified, and a methanol-only control group was not included. A separate 0.5% CMC vehicle-control group without pesticide or botanical extract was also not included. Consequently, the independent effect of CMC on mortality or locomotor performance cannot be distinguished from the effects of pesticide or phytotherapeutic exposure in the present experimental design.

### 2.5 Experimental organism and fly husbandry

Wild-type *Drosophila melanogaster* (Oregon K strain) were obtained from the *Drosophila* stock centre, Department of Zoology, University of Dar es Salaam, Tanzania. Flies were maintained on wheat–agar culture medium under controlled laboratory conditions at 22 °C ± 1 °C, 60%–70% relative humidity, and a 12:12 h light/dark cycle (Pandey and N., 2011). Only adult male flies aged 4–7 days were used throughout the study. Young adult flies were selected to provide a relatively stable locomotor baseline while minimizing v °ariability associated with age-dependent deterioration in negative-geotaxis performance (Pandey and N., 2011). Negative geotaxis is known to decline with advancing age in *Drosophila* (Madabattula et al., 2015), and age-related physiological and behavioural changes can introduce substantial variability into experimental phenotypes (Wodrich et al., 2023). The 4–7-day age window was therefore used to minimize confounding by natural locomotor senescence while ensuring that fully developed adult flies were evaluated. Male flies were used consistently to reduce variability associated with sex and reproductive state (Pandey and N., 2011).

### 2.6 *Mucuna pruriens* toxicity assessment

Adult male *D. melanogaster* were allocated to five experimental groups: a negative-control group receiving standard fly diet only and four treatment groups receiving diets containing 0.1%, 0.5%, 1.0%, or 2.0% (w/v) crude *M. pruriens* seed extract prepared in 0.5% (w/v) CMC. The experiment was conducted in three independent experimental phases. Within each phase, each treatment was represented by three separate vials containing 20 flies per vial, giving nine vial-level experimental units and 180 flies per treatment. Flies remained continuously on the assigned diets for seven consecutive days. Mortality was recorded every 24 h, and dead flies were removed after each observation. Daily cumulative mortality, seven-day endpoint mortality, survival probability, mortality hazard, and concentration–response parameters were subsequently determined.

### 2.7 Preliminary pesticide mortality and selection of induction concentrations

Before locomotor testing, seven-day mortality was assessed following dietary exposure to paraquat at 10, 20, 30, and 40 mM and glyphosate at 0.5%, 1.0%, and 2.0% (w/v). All pesticide preparations contained 0.5% (w/v) CMC as the vehicle. Each treatment was evaluated in three independent experimental phases, with three vials per phase and 20 flies per vial, resulting in nine vials and 180 flies per treatment. Paraquat concentrations of 10 and 20 mM were selected for subsequent behavioural experiments because they produced measurable toxic stress while retaining sufficient survival, whereas mortality exceeded 50% at 30 mM and increased further at 40 mM. All three glyphosate concentrations were retained because survival remained sufficient for locomotor assessment.

### 2.8 Induction of pesticide-associated locomotor impairment

Adult male *D. melanogaster* were exposed for seven consecutive days to diets containing paraquat at 10 or 20 mM or glyphosate at 0.5%, 1.0%, or 2.0% (w/v). Each pesticide preparation contained 0.5% (w/v) CMC as the vehicle. The negative-control group received standard fly diet only, whereas pesticide-only groups received the corresponding pesticide-containing diet without botanical extract. Fresh exposure media were prepared regularly, and flies were transferred to freshly prepared media at approximately 48-h intervals. The pesticide concentrations were selected to induce reproducible locomotor impairment while maintaining sufficient survival for subsequent behavioural assessment (OECD, 2001). They were therefore experimental model-induction concentrations and were not intended to reproduce environmentally or occupationally relevant human exposure levels.

### 2.9 Phytotherapeutic formulations and treatment

Following the pesticide-exposure period, flies assigned to phytotherapeutic treatment groups received diets containing 0.1% (w/v) *M. pruriens* alone or *M. pruriens* in combination with 0.1% *C. pepo* and/or 0.1% *A. hypogaea*. All botanical extracts were suspended in 0.5% (w/v) CMC before incorporation into the diet. The 0.1% *M. pruriens* concentration was selected because it corresponded to the operational NOEL identified in the preceding toxicity assessment. *Mucuna pruriens* monotherapy contained 0.1% total crude extract, binary formulations contained 0.2%, and the ternary formulation contained 0.3%. Treatment diets were administered for seven consecutive days before final locomotor assessment. Because total crude-extract concentration differed among formulations, numerical differences cannot be interpreted as evidence of additive or synergistic pharmacological interactions.

### 2.10 Negative geotaxis assay

Locomotor performance was evaluated using the negative geotaxis assay in three independent experimental runs (Aggarwal et al., 2019). Within each run, every treatment was represented by four separate vials containing 10 male flies per vial. Each treatment therefore comprised 12 independent vial-level experimental units and 120 flies in total. The vial was considered the experimental unit, while individual flies within each vial were treated as subsamples. For assessment, flies were transferred into clean vertical assay tubes and allowed to acclimatize before being gently tapped to the bottom. Climbing performance was recorded within 10 s using the 5-cm climbing criterion specified in the experimental protocol (Madabattula et al., 2015). Failure to reach the 5-cm threshold within 10 s was interpreted as impaired negative geotaxis. Locomotor response was additionally expressed using the normalized assay scale employed in the study, with 1.0 representing the normal reference and 0.5 representing the prespecified threshold for marked locomotor impairment. Because dopaminergic neuronal integrity and neurochemical biomarkers were not assessed, this phenotype was interpreted as Parkinsonism-like locomotor impairment rather than definitive Parkinson’s disease pathology. Each vial contributed ten ordered observations, designated L1–L10. These observations represented repeated measurements from the same vial and were therefore not considered independent biological replicates.

### 2.11 Locomotor performance and recovery metrics

For each vial, locomotor performance was summarized using the area under the climbing-response curve (AUC) across the ten ordered observations (L1–L10). Because adjacent L1–L10 observations were treated as equally spaced ordered assay measurements, vial-level AUC was calculated using the trapezoidal rule as:

$$\text{AUC}=\sum_{i=1}^{9}\left[\frac{R_{i}+R_{i+1}}{2}\right]\times \left(x_{i+1}-x_{i}\right)$$

where $R_{i}$ and $R_{i+1}$ are consecutive locomotor responses recorded at adjacent ordered observations (L i and L i+1 ), and $x_{i}$ and $x_{i+1}$ are the corresponding ordered observation indices (equally spaced). For the equally spaced L1–L10 measurements, $\left(x_{i+1}-x_{i}\right)$ is constant, and AUC therefore reflects the overall level and persistence of responses across L1–L10. AUC was calculated independently for each vial and was used as the primary locomotor endpoint. Higher AUC values indicated better and more sustained locomotor performance across L1–L10.

#### 2.11.1 Performance relative to the negative control

For descriptive comparisons among treatment groups, group-level performance relative to the negative control was calculated from the group mean vial-level AUC as:

$$\text{Performance relative to control }\left(\%\right)=100\times \frac{{\text{AUC}}_{\mathrm{group}}}{{\text{AUC}}_{NC}}$$

where ${\text{AUC}}_{\text{group}}$ is the mean AUC of the treatment or pesticide group and ${\text{AUC}}_{NC}$ is the mean AUC of the negative-control group.

#### 2.11.2 Pesticide-associated locomotor impairment

Pesticide-associated locomotor impairment relative to the negative control was calculated as:

$$\text{Locomotor impairment }\left(\%\right)=100-\text{Performance relative to control }\left(\%\right)$$

Thus, 0% indicated performance equivalent to the negative-control reference, whereas higher values indicated greater impairment relative to the negative control.

#### 2.11.3 Relative improvement after phytotherapeutic treatment

For phytotherapeutic treatment groups, relative improvement was calculated as:

$$\text{Relative improvement }\left(\%\right)=100\times \frac{{\text{AUC}}_{treated}-{\text{AUC}}_{pesticide}}{{\text{AUC}}_{pesticide}}$$

where ${\text{AUC}}_{treated}$ is the mean AUC following phytotherapeutic treatment and ${\text{AUC}}_{pesticide}$ is the mean AUC of the corresponding pesticide-only group.

A value of 0% indicated no improvement over the pesticide-only baseline, whereas positive values indicated increased locomotor performance following treatment.

#### 2.11.4 Recovery toward normal locomotor performance

Recovery toward normal was calculated as:

$$\text{Recovery toward normal }\left(\%\right)=100\times \frac{{\text{AUC}}_{treated}-{\text{AUC}}_{pesticide}}{{\text{AUC}}_{NC}-{\text{AUC}}_{pesticide}}$$

Under this definition, 0% represented no improvement beyond the pesticide-only baseline, whereas 100% represented attainment of the negative-control mean AUC. Values below 0% indicated poorer performance than the pesticide-only baseline, while values above 100% indicated performance exceeding the negative-control reference. The normal range across L1–L10 was operationally defined as a treatment-group mean locomotor response equal to or greater than the lower 95% confidence limit of the negative-control response at the corresponding observation. The first normal-range reading was defined as the earliest L1–L10 observation satisfying this criterion. The first sustained normal-range reading was the earliest observation at which the criterion was satisfied and remained satisfied for all subsequent observations through L10. Where neither criterion was achieved, the result was recorded as “Not reached.” Because L1–L10 were ordered assay observations rather than independently measured clock-time points, these endpoints were not interpreted as recovery times. For recovery calculations in which treatment and pesticide-only reference values originated from separate assay datasets, the resulting percentages were considered descriptive metrics and not direct inferential treatment comparisons.

### 2.12 Statistical analysis

Statistical analyses were performed using R version 4.5.3. The vial was considered the experimental unit for all inferential analyses. Individual flies within each vial were treated as subsamples, and repeated L1–L10 observations from the same vial were not considered independent biological replicates. For mortality analyses, deaths and mortality proportions were summarized with exact 95% binomial confidence intervals. Overall differences in seven-day mortality among M. pruriens concentrations were evaluated using Pearson’s chi-square test, and a one-degree-of-freedom concentration-trend test was used to assess the concentration-related mortality pattern. Pairwise, mortality comparisons relative to the negative control were expressed as risk ratios with 95% confidence intervals, with p-values adjusted for multiple comparisons using the Holm method. Time-to-event survival was evaluated using Kaplan-Meier analysis, and overall survival distributions were compared using the log-rank test. Cox proportional-hazards regression was used to estimate hazard ratios and 95% confidence intervals relative to the negative control, with vial-level clustering incorporated into variance estimation. Kaplan-Meier analysis was used to complement endpoint mortality by accounting for both the occurrence and timing of deaths during the seven-day exposure period. The concentration–mortality relationship for M. pruriens was modelled using logistic and probit regression. Median lethal concentration (LC_50_) estimates and corresponding 95% confidence intervals were derived from the fitted models. Because mortality remained below 50% at the highest experimentally administered concentration of 2.0% (w/v), LC_50_ estimates above 2.0% were interpreted as model-dependent extrapolations rather than directly observed or interpolated toxicity thresholds. Model fit was additionally summarized using the Akaike information criterion (AIC).

For locomotor analyses, AUC was calculated independently for each vial across L1–L10 and used as the primary inferential endpoint. Treatment effects were assessed using a blocked linear model:

$$AUC_{ij}=\mu +Treatment_{i}+Phase_{j}+\epsilon_{ij}$$

which was specified in R as:

AUC∼Treatment+Phase

where Treatment represented the experimental treatment factor and Phase represented the experimental blocking factor. Estimated marginal means were obtained from the fitted models, and pairwise comparisons were adjusted for multiple testing using Tukey’s method. Where appropriate, Kruskal–Wallis tests followed by pairwise Wilcoxon rank-sum comparisons were conducted as non-parametric sensitivity analyses. Locomotor trajectories across L1–L10 were summarized descriptively using means and standard errors. Because L1–L10 represented repeated observations within the same vial, they were not analysed as independent biological replicates, and inferential conclusions were based primarily on vial-level AUC. All statistical tests were two-sided, and statistical significance was defined as p<0.05 .

## 3 Results

### 3.1 Concentration-dependent toxicity of *Mucuna pruriens*

#### 3.1.1 Day-wise mortality and endpoint toxicity

**FIGURE 1:**
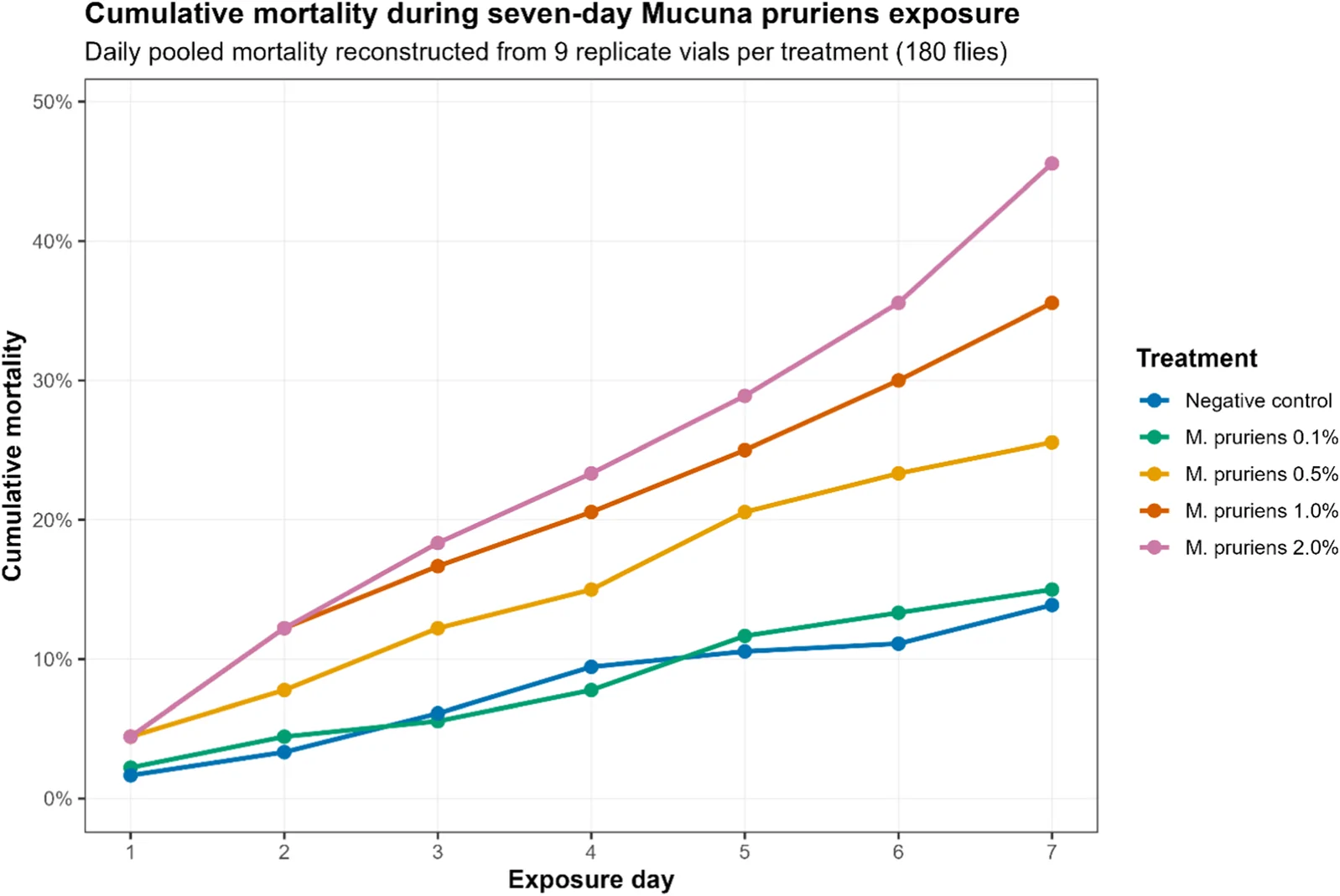
Cumulative mortality of adult *Drosophila melanogaster* during seven-day dietary exposure to crude *Mucuna pruriens* seed extract. Curves show cumulative mortality across days 1–7 for the negative control and 0.1%, 0.5%, 1.0%, and 2.0% (w/v) M. pruriens. Each treatment initially comprised 180 flies distributed among nine replicate vials. Mortality increased progressively with exposure duration and extract concentration.

Mortality increased progressively during the seven-day dietary exposure period and showed a clear concentration-dependent pattern (Figure 1). On day 7, mortality was 13.9% in the negative control and 15.0%, 25.6%, 35.6%, and 45.6% following exposure to 0.1%, 0.5%, 1.0%, and 2.0% (w/v) *Mucuna pruriens*, respectively. Corresponding survival decreased from 86.1% in the negative control to 54.4% at the highest tested concentration (Table 1). Mortality differed significantly among treatment groups (Pearson χ^2^ = 66.99, df = 4, *p* < 0.001). A significant concentration-related trend was also observed (χ^2^ = 64.14, df = 1, *p* < 0.001), indicating progressively greater mortality with increasing *M. pruriens* concentration.

**TABLE 1:** Seven-day endpoint mortality and survival of adult *Drosophila melanogaster* following dietary exposure to crude *Mucuna pruriens* seed extract.

Treatment	Concentration (% w/v)	Vials (n)	Flies (n)	Deaths (n)	Mortality, % (95% CI)	Survival (%)
Negative control	0	9	180	25	13.9 (9.2–19.8)	86.1
*M. pruriens*	0.1	9	180	27	15.0 (10.1–21.1)	85.0
*M. pruriens*	0.5	9	180	46	25.6 (19.4–32.6)	74.4
*M. pruriens*	1.0	9	180	64	35.6 (28.6–43.0)	64.4
*M. pruriens*	2.0	9	180	82	45.6 (38.1–53.1)	54.4

Each treatment comprised three independent experimental phases, with three vials per phase and 20 flies per vial, giving nine vials and 180 flies per treatment. Mortality represents cumulative deaths recorded during the seven-day exposure period.

#### 3.1.2 Mortality risk, survival probability, NOEL and LOEL

**FIGURE 2:**
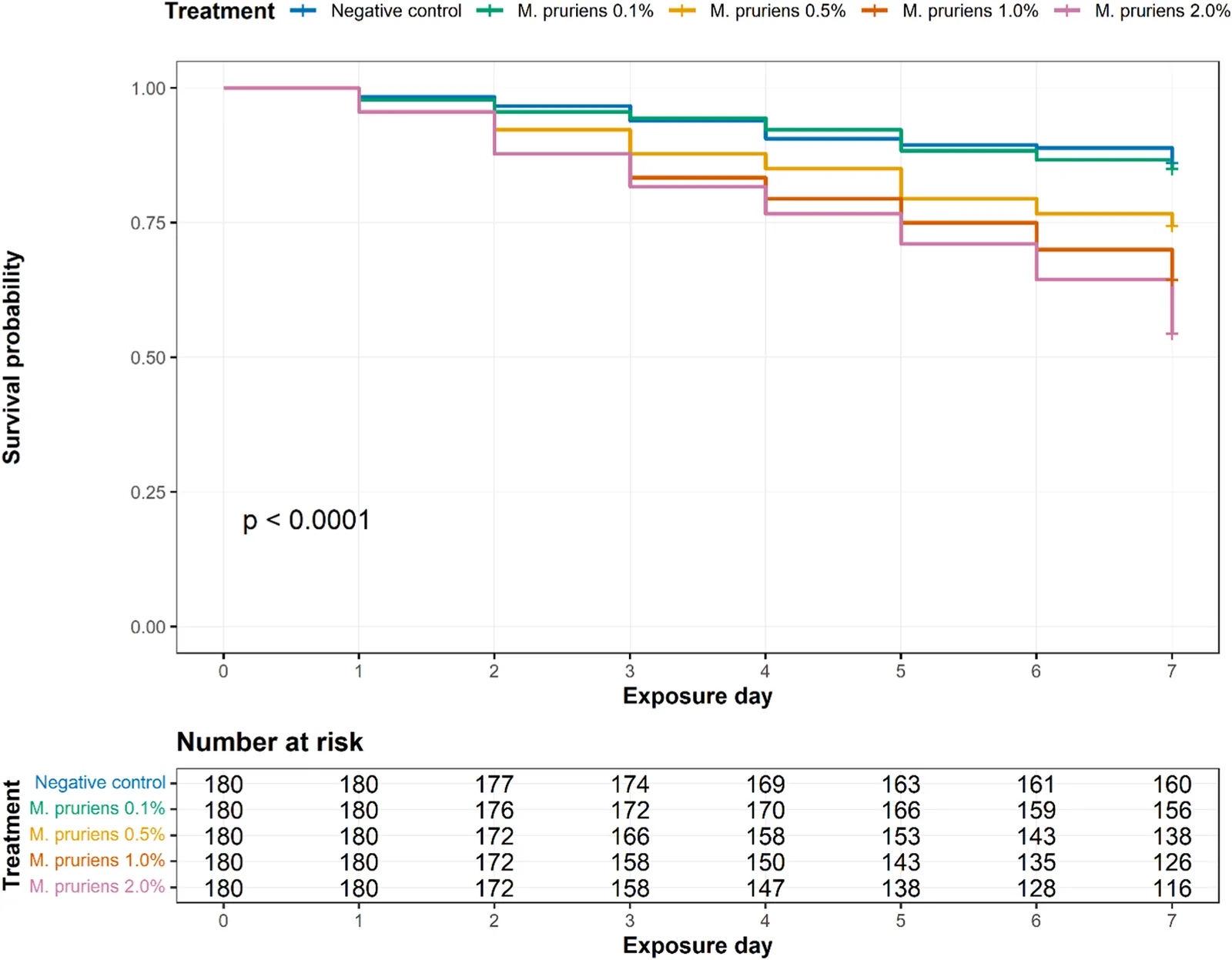
Kaplan–Meier survival curves of adult *Drosophila melanogaster* during seven-day dietary exposure to crude Mucuna pruriens seed extract. Survival probability declined progressively with increasing extract concentration. Kaplan-Meier analysis complements endpoint mortality by incorporating the timing of deaths throughout the seven-day exposure period. Overall survival distributions differed significantly among treatment groups (log-rank χ^2^ = 65.67, df = 4, p < 0.001).

Seven-day mortality at 0.1% *M. pruriens* was comparable with the negative control (RR = 1.08, 95% CI: 0.65–1.79; Holm-adjusted *p* = 0.881). In contrast, mortality risk increased significantly at 0.5% (RR = 1.84, 95% CI: 1.18–2.86; Holm-adjusted *p* = 0.016), 1.0% (RR = 2.56, 95% CI: 1.69–3.87; *p* < 0.001), and 2.0% (RR = 3.28, 95% CI: 2.20–4.88; *p* < 0.001) relative to the negative control (Table 2). Accordingly, 0.1% was identified as the operational NOEL, while 0.5% represented the LOEL under the present seven-day experimental conditions. Kaplan–Meier survival distributions differed significantly among groups (log-rank χ^2^ = 65.67, df = 4, *p* < 0.001; Figure 2). Relative to the negative control, the mortality hazard was similar at 0.1% but increased approximately twofold at 0.5%, threefold at 1.0%, and fourfold at 2.0% *M. pruriens* (Table 2). Unlike seven-day endpoint mortality, which summarizes only the final proportion of deaths, Kaplan-Meier analysis incorporates both whether death occurred and when it occurred during the exposure period. It therefore provides complementary information on the temporal pattern of mortality.

**TABLE 2:** Mortality risk and survival hazard following seven-day dietary exposure to crude *Mucuna pruriens* seed extract.

*M. pruriens* concentration	Risk ratio (95% CI)	Holm-adjusted *p*	Hazard ratio (95% CI)	Cox *p*-value	Toxicological interpretation
0.1%	1.08 (0.65–1.79)	0.881	1.09 (0.69–1.70)	0.716	Operational NOEL
0.5%	1.84 (1.18–2.86)	0.016	1.99 (1.39–2.87)	<0.001	LOEL
1.0%	2.56 (1.69–3.87)	<0.001	2.94 (2.13–4.05)	<0.001	Increased toxicity
2.0%	3.28 (2.20–4.88)	<0.001	3.91 (2.83–5.40)	<0.001	Highest tested toxicity

Risk ratios and hazard ratios are expressed relative to the negative control. NOEL, no-observed-effect level; LOEL, lowest-observed-effect level. The classifications apply specifically to the seven-day experimental conditions used in this study.

#### 3.1.3 Concentration–response modelling and LC_50_

**FIGURE 3:**
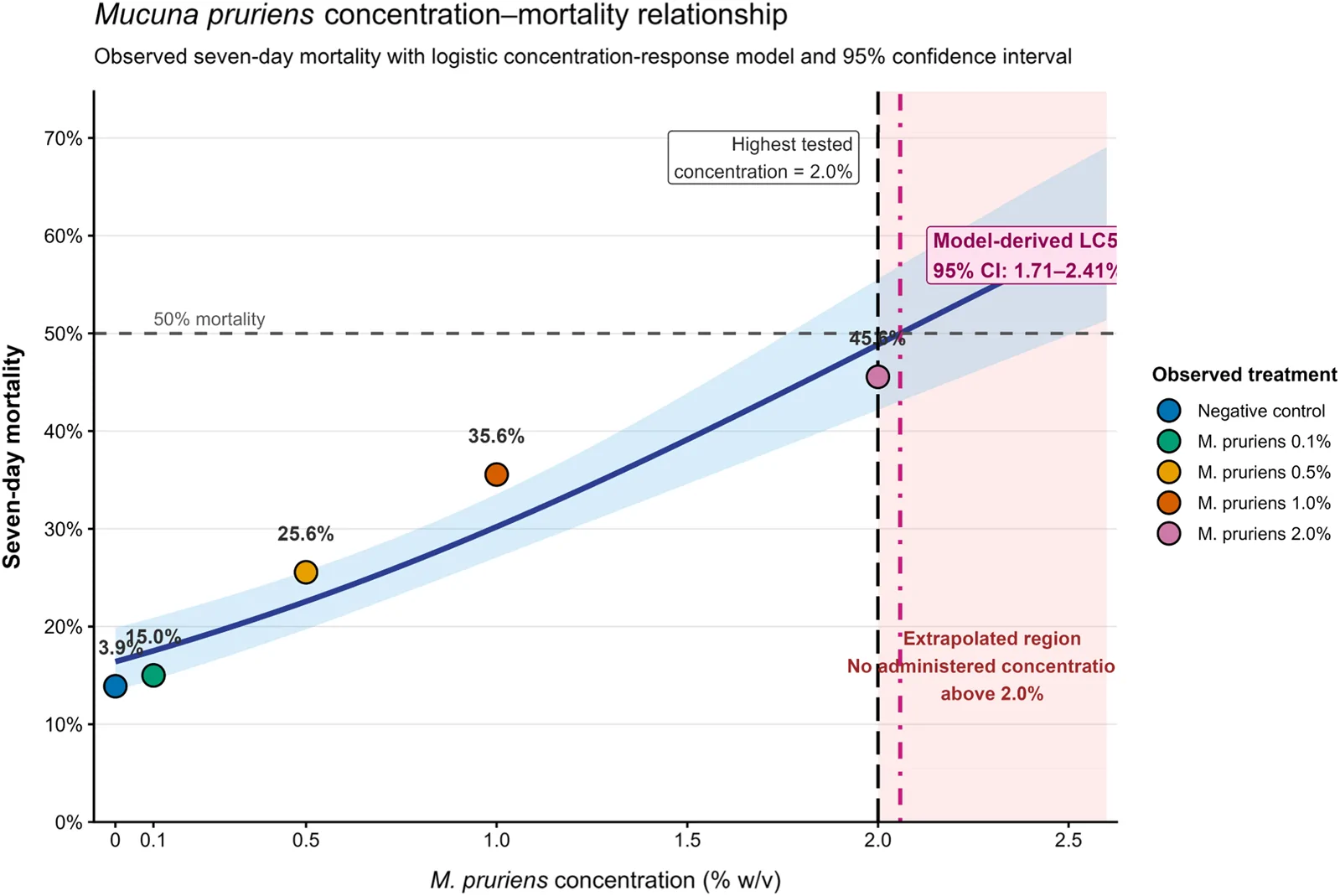
Modelled concentration–mortality relationship for crude Mucuna pruriens seed extract following seven-day dietary exposure. Points represent observed seven-day mortality at the negative control and 0.1%, 0.5%, 1.0%, and 2.0% (w/v) M. pruriens. The fitted curve represents the logistic concentration–response model, with the shaded band indicating the 95% confidence interval. The horizontal dashed line represents 50% mortality, while the vertical line at 2.0% identifies the highest experimentally administered concentration. The region beyond 2.0% represents extrapolation. The model-derived LC50 was approximately 2.06% (95% CI: approximately 1.71%–2.41%) and should be interpreted cautiously because 50% mortality was not directly observed.

Logistic and probit concentration–response models generated closely comparable LC_50_ estimates (Table 3; Figure 3). The logistic model estimated an LC_50_ of 2.058% (95% CI: 1.705%–2.411%), whereas the probit model estimated an LC_50_ of 2.061% (95% CI: 1.700%–2.423%). The similarity of the estimates indicates consistency between the two fitted modelling approaches. However, mortality at the highest experimentally administered concentration of 2.0% was 45.6%; therefore, the 50% mortality level was not directly observed within the tested concentration range. LC_50_ values consequently represent model-dependent extrapolations beyond the highest experimentally administered concentration rather than interpolations based on observed mortality values on both sides of 50%. The estimates should therefore be interpreted as approximate model-derived concentration–response parameters rather than precisely established experimental toxicity thresholds. Future toxicity studies should include concentrations above 2.0% to provide empirical observations around and above 50% mortality and more securely characterize the upper portion of the concentration–response curve.

**TABLE 3:** Model-derived LC_50_ estimates for crude *Mucuna pruriens* seed extract following seven-day dietary exposure.

Model	LC_50_ (% w/v)	95% CI (% w/v)	Maximum tested concentration (%)	Extrapolated	AIC
Logistic	2.058	1.705–2.411	2.0	Yes	36.20
Probit	2.061	1.700–2.423	2.0	Yes	35.55

Mortality remained below 50% at the highest experimentally administered concentration of 2.0% (w/v). Consequently, both LC_50_ estimates are model-dependent extrapolations beyond the tested concentration range. AIC, akaike information criterion; LC_50_, model-estimated concentration associated with 50% mortality.

### 3.2 Preliminary pesticide mortality and selection of locomotor-model concentrations

**FIGURE 4:**
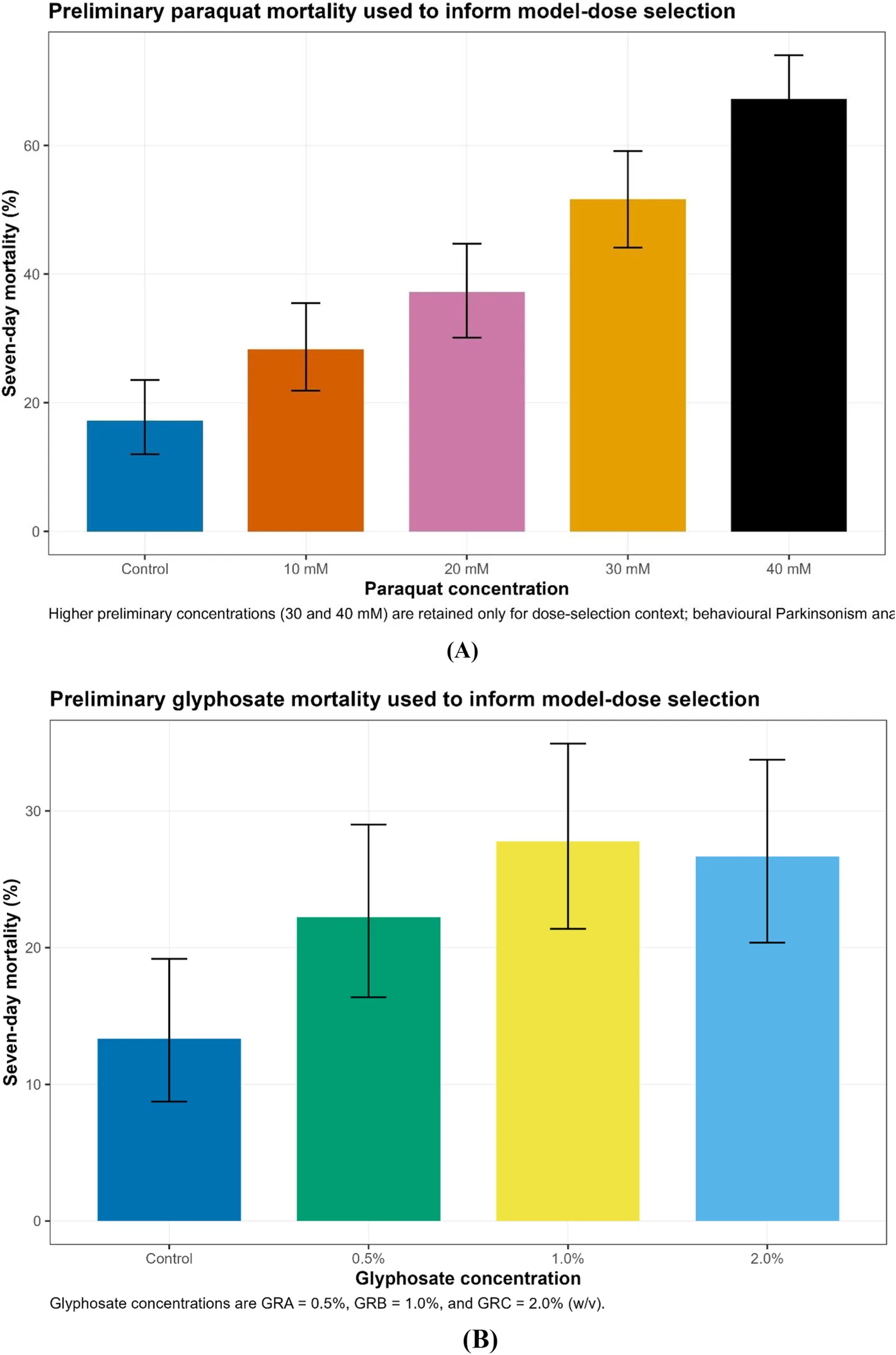
Preliminary seven-day pesticide mortality used to inform experimental concentration selection. **(A)** Paraquat mortality increased from 28.3% at 10 mM to 67.2% at 40 mM. Mortality exceeded 50% at 30 mM, supporting selection of 10 and 20 mM for subsequent locomotor experiments. **(B)** Glyphosate mortality at 0.5%, 1.0%, and 2.0% (w/v) was 22.2%, 27.8%, and 26.7%, respectively, with survival remaining above 70% at all three concentrations. Error bars represent 95% confidence intervals.

Preliminary seven-day mortality screening was conducted to identify pesticide concentrations that produced measurable toxic stress while retaining sufficient survival for subsequent locomotor assessment. Paraquat showed a clear concentration-dependent increase in mortality, rising from 28.3% at 10 mM to 37.2% at 20 mM, 51.7% at 30 mM, and 67.2% at 40 mM (Table 4; Figure 4A). Corresponding survival declined from 71.7% at 10 mM to 32.8% at 40 mM. Because mortality exceeded 50% at 30 mM and increased further at 40 mM, these higher concentrations were not retained for behavioural modelling. Paraquat concentrations of 10 and 20 mM were selected because they produced substantial pesticide-associated stress while preserving 71.7% and 62.8% survival, respectively, thereby allowing subsequent locomotor assessment. Glyphosate produced mortality of 22.2%, 27.8%, and 26.7% at 0.5%, 1.0%, and 2.0% (w/v), respectively (Table 4; Figure 4B). Survival remained above 70% at all three concentrations. Unlike paraquat, glyphosate mortality did not show a strictly monotonic increase across the tested concentrations, because mortality at 2.0% was slightly lower than at 1.0%. Nevertheless, survival remained sufficient at all three concentrations to permit behavioural evaluation. Therefore, 0.5%, 1.0%, and 2.0% (w/v), designated GRA, GRB, and GRC, respectively, were retained as experimental locomotor-model concentrations. These concentrations were selected empirically for the experimental fly model and should not be interpreted as equivalent to occupational, dietary, residential, or environmental human exposure levels.

**TABLE 4:** Preliminary seven-day pesticide mortality used to inform selection of locomotor-model concentrations.

Pesticide	Concentration	Flies (n)	Deaths (n)	Mortality, % (95% CI)	Survival (%)	Selection for locomotor assay
Paraquat control	—	180	31	17.2 (12.0–23.5)	82.8	References
Paraquat	10 mM	180	51	28.3 (21.9–35.5)	71.7	Selected
Paraquat	20 mM	180	67	37.2 (30.1–44.7)	62.8	Selected
Paraquat	30 mM	180	93	51.7 (44.1–59.2)	48.3	Not selected
Paraquat	40 mM	180	121	67.2 (59.8–74.0)	32.8	Not selected
Glyphosate control	—	180	24	13.3 (8.7–19.2)	86.7	References
Glyphosate	0.5% (w/v)	180	40	22.2 (16.4–29.0)	77.8	Selected
Glyphosate	1.0% (w/v)	180	50	27.8 (21.4–34.9)	72.2	Selected
Glyphosate	2.0% (w/v)	180	48	26.7 (20.4–33.8)	73.3	Selected

Each treatment comprised three independent experimental phases, with three vials per phase and 20 flies per vial, giving nine vials and 180 flies per treatment. Paraquat 10 and 20 mM were selected because mortality remained below 50%, whereas mortality exceeded 50% at 30 mM. All glyphosate concentrations were retained because survival remained above 70%. Pesticide preparations contained 0.5% (w/v) CMC, as vehicle. The control groups shown are those used in the corresponding preliminary pesticide assays.

### 3.3 Pesticide-associated locomotor impairment

**FIGURE 5:**
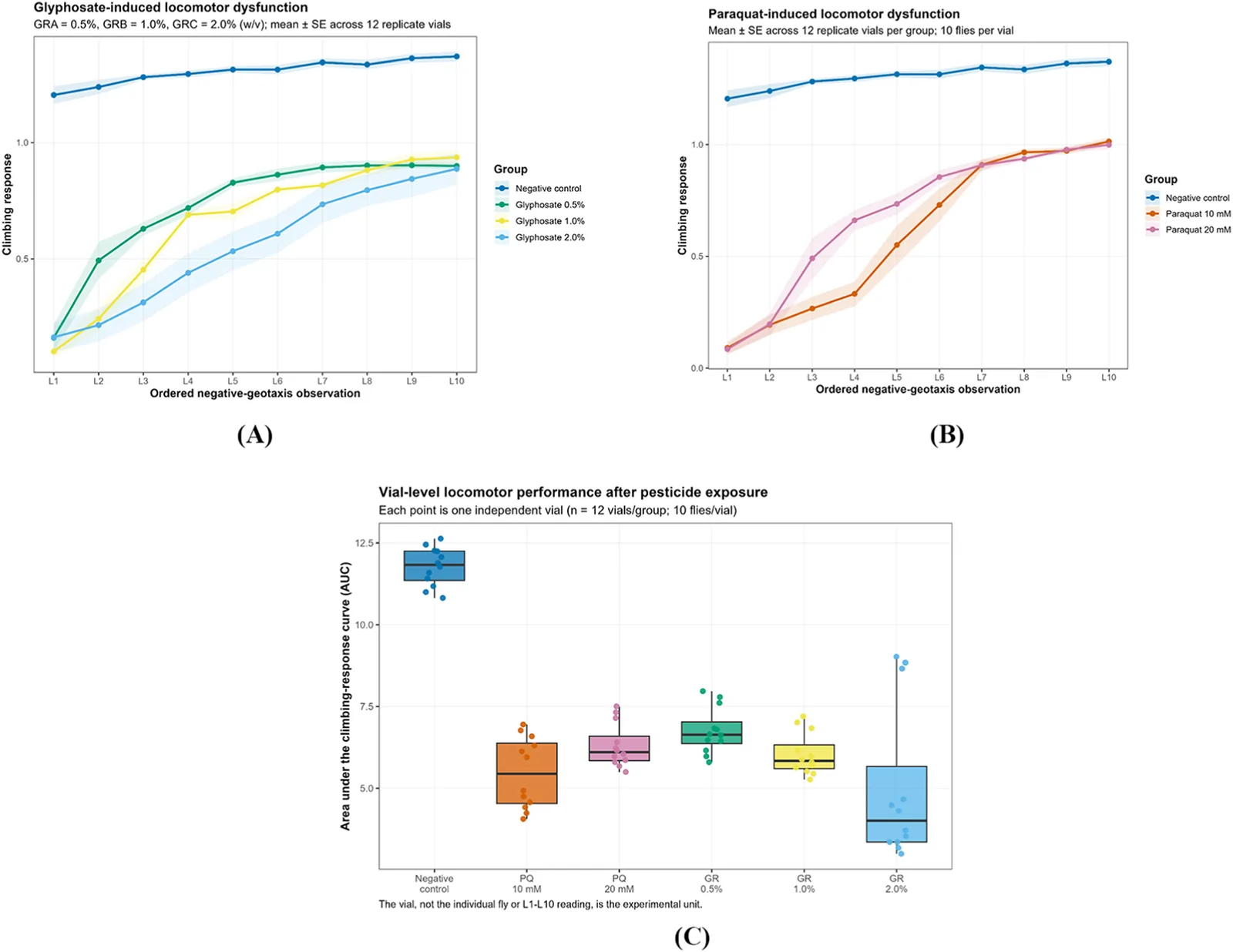
Pesticide-associated locomotor impairment in adult *Drosophila melanogaster*. **(A)** Mean ± SE negative-geotaxis trajectories across L1–L10 following glyphosate exposure at 0.5%, 1.0%, and 2.0% (w/v). **(B)** Mean ± SE trajectories following paraquat exposure at 10 and 20 mM. **(C)** Vial-level AUC distributions for negative control and all pesticide-exposed groups. The negative control represents flies maintained on standard diet, whereas the pesticide-only groups represent the corresponding pesticide-associated locomotor-impairment baselines. Each point in panel C represents one independent vial containing 10 flies; n = 12 vials per treatment. Distinct colours identify the negative control and each pesticide concentration. Inferential comparisons were based on vial-level AUC rather than individual flies or repeated L1–L10 observations.

Paraquat and glyphosate exposure substantially reduced vial-level locomotor performance relative to the negative control (Table 5; Figure 5). The blocked AUC model demonstrated a significant overall treatment effect (*p* < 0.001), whereas experimental phase did not significantly affect AUC (*p* = 0.583). Following Tukey adjustment, all pesticide-exposed groups had significantly lower vial-level AUC than the negative control (all adjusted *p* < 0.001). The negative-control group had a mean AUC of 11.779 ± 0.587. Glyphosate exposure produced a concentration-related reduction in locomotor performance, with mean AUC decreasing from 6.759 ± 0.700 at 0.5% to 6.033 ± 0.644 at 1.0% and 5.009 ± 2.369 at 2.0% (w/v). Performance relative to the negative control was 57.4%, 51.2%, and 42.5%, respectively, corresponding to locomotor impairment of 42.6%, 48.8%, and 57.5%. Paraquat also produced substantial locomotor impairment. Mean AUC was 5.472 ± 1.074 at 10 mM and 6.301 ± 0.668 at 20 mM, corresponding to performance levels of 46.5% and 53.5% and locomotor impairment values of 53.5% and 46.5%, respectively. Although mean AUC was numerically lower at 10 mM than at 20 mM, a targeted phase-adjusted comparison (difference = −0.829, SE = 0.377; nominal *p* = 0.040) was not significant after Tukey adjustment within the full six-group model (adjusted *p* = 0.538). Thus, both paraquat concentrations significantly impaired locomotor performance relative to the negative control, but the data did not establish a monotonic paraquat concentration–response relationship.

**TABLE 5:** Vial-level locomotor performance following paraquat or glyphosate exposure.

Treatment	Vials (n)	Flies (n)	Mean response ± SD	Mean AUC ± SD	Performance relative to negative control (%)	Locomotor impairment relative to negative control (%)
Negative control	12	120	1.307 ± 0.068	11.779 ± 0.587	100.0	0.0
Paraquat 10 mM	12	120	0.602 ± 0.109	5.472 ± 1.074	46.5	53.5
Paraquat 20 mM	12	120	0.684 ± 0.065	6.301 ± 0.668	53.5	46.5
Glyphosate 0.5% (w/v)	12	120	0.729 ± 0.081	6.759 ± 0.700	57.4	42.6
Glyphosate 1.0% (w/v)	12	120	0.655 ± 0.073	6.033 ± 0.644	51.2	48.8
Glyphosate 2.0% (w/v)	12	120	0.553 ± 0.259	5.009 ± 2.369	42.5	57.5

Each treatment comprised three independent experimental runs, with four vials per treatment per run and 10 male flies per vial, giving 12 independent vial-level experimental units and 120 flies per treatment. The vial was the experimental unit. Performance relative to negative control (%) was calculated as: $100\times \frac{AUC_{group}}{AUC_{NC}}$ , Locomotor impairment relative to negative control (%) was calculated as: $100-performance\;relative\;to\;negative\;control\;\left(\%\right)$ and Inferential comparisons were conducted using vial-level AUC.

### 3.4 Locomotor improvement following 0.1% *Mucuna pruriens* monotherapy

**FIGURE 6:**
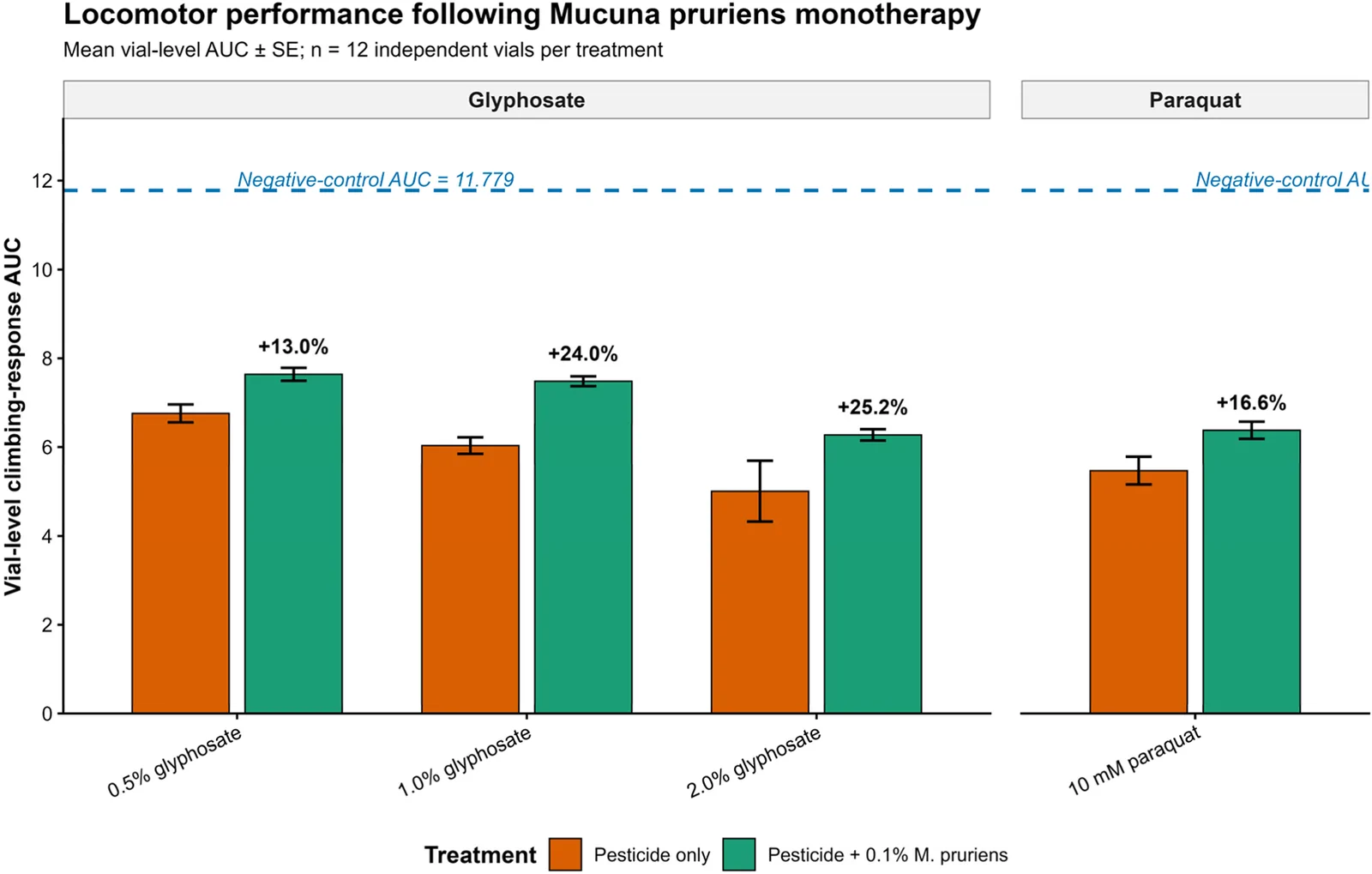
Locomotor performance following 0.1% *Mucuna pruriens* treatment after pesticide exposure. Mean vial-level area under the climbing-response curve (AUC) ± SE is shown for pesticide-only and M. pruriens-treated groups following glyphosate exposure at 0.5%, 1.0%, and 2.0% (w/v) and paraquat exposure at 10 mM. Each group comprised 12 independent vials containing 10 flies per vial. The dashed horizontal line represents the mean negative-control AUC (11.779). Percentages above the treated groups indicate relative improvement compared with the corresponding pesticide-only baseline. M. pruriens treatment was associated with partial numerical locomotor improvement, but all treated groups remained below the negative-control reference.

Treatment with 0.1% *Mucuna pruriens* was associated with numerical improvement in locomotor performance relative to the corresponding pesticide-only baselines (Table 6; Figure 6). Following 0.5%, 1.0%, and 2.0% glyphosate exposure, mean AUC increased from pesticide-only values of 6.759, 6.033, and 5.009 to 7.640, 7.483, and 6.273, respectively, following *M. pruriens* treatment. These changes corresponded to relative improvements of 13.0%, 24.0%, and 25.2%, respectively. When expressed relative to the negative-control AUC, locomotor performance following *M. pruriens* treatment was 64.9%, 63.5%, and 53.3% after exposure to 0.5%, 1.0%, and 2.0% glyphosate, respectively. Recovery toward normal locomotor performance was 17.6%, 25.2%, and 18.7%, respectively. Thus, although treatment improved performance relative to the corresponding pesticide-only baselines, recovery remained substantially below the negative-control reference. In the 10 mM paraquat model, mean AUC increased numerically from 5.472 in the pesticide-only group to 6.378 following 0.1% *M. pruriens* treatment. This represented a relative improvement of 16.6%, while performance reached 54.1% of the negative-control reference. Recovery toward normal was 14.4%. Overall, recovery toward normal ranged from 14.4% to 25.2% across the pesticide models. These findings indicate partial treatment-associated locomotor improvement rather than normalization of locomotor function. The blocked analysis of the combination-treatment dataset demonstrated a significant overall treatment effect (*p* < 0.001), whereas the effect of experimental phase was not significant (*p* = 0.387). Among the 2.0% glyphosate combination groups, the ternary formulation had a significantly higher AUC than *M. pruriens* + *C. pepo* after Tukey adjustment (adjusted *p* = 0.008). *Mucuna pruriens* + *A. hypogaea* did not differ significantly from either formulation. No pairwise differences among the three paraquat combination formulations remained significant after Tukey adjustment the corresponding groups treated with 0.1% *M. pruriens*. Error bars are SE calculated from 12 independent vials, not from 120 individual flies. The dashed blue line represents the negative-control AUC of 11.779. The percentages above the *M. pruriens* bars show the descriptive relative improvement over the corresponding pesticide-only baseline.

**TABLE 6:** Locomotor performance following 0.1% *Mucuna pruriens* treatment after pesticide exposure.

Pesticide exposure	Pesticide-only AUC	Locomotor impairment relative to negative control (%)	MP-treated AUC	MP performance relative to negative control (%)	Relative improvement (%)	Recovery toward normal (%)
Glyphosate 0.5% (w/v)	6.759	42.6	7.640	64.9	13.0	17.6
Glyphosate 1.0% (w/v)	6.033	48.8	7.483	63.5	24.0	25.2
Glyphosate 2.0% (w/v)	5.009	57.5	6.273	53.3	25.2	18.7
Paraquat 10 mM	5.472	53.5	6.378	54.1	16.6	14.4

MP refers to Mucuna pruriens. Pesticide-associated locomotor impairment was calculated relative to the negative-control AUC. Relative improvement (%) was calculated as: $100\times \frac{\left(AUC_{treated}-AUC_{pesticide}\right)}{AUC_{pesticide}},$ Recovery toward normal (%) was calculated as: $100\times \frac{\left(AUC_{treated}-AUC_{pesticide}\right)}{\left(AUC_{NC}-AUC_{pesticide}\right)}$ , A recovery value of 0% indicates no improvement above the pesticide-only baseline, whereas 100% indicates attainment of the negative-control AUC. these recovery measures are descriptive and should not be interpreted as evidence of neuronal restoration or a specific neuroprotective mechanism.

### 3.5 Locomotor outcomes following combination phytotherapy

**FIGURE 7:**
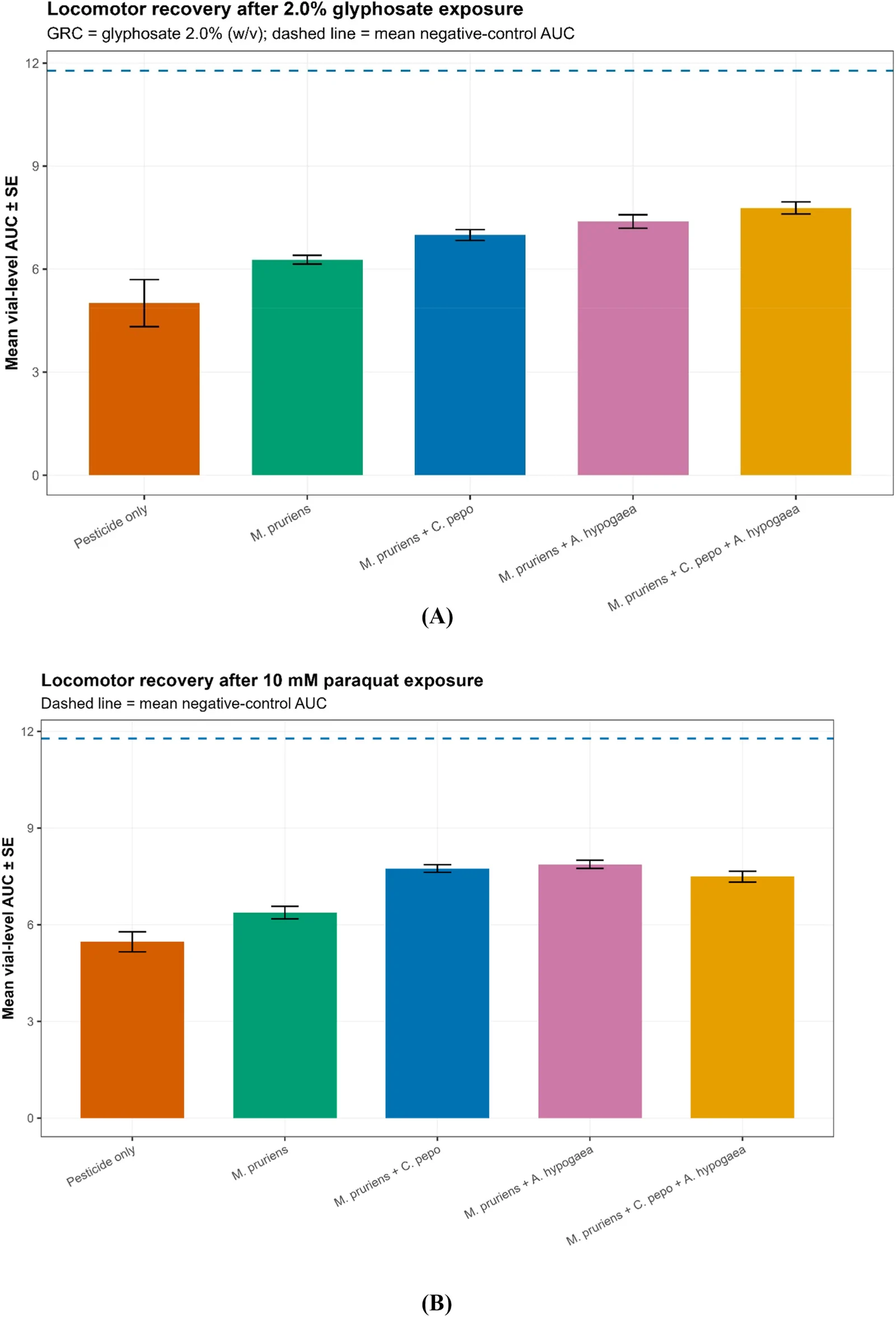
Locomotor outcomes following *Mucuna pruriens*-based combination treatment after pesticide exposure. **(A)** Mean ± SE vial-level AUC following 2.0% (w/v) glyphosate exposure and subsequent treatment with M. pruriens alone or M. pruriens-based binary and ternary formulations. **(B)** Corresponding mean ± SE vial-level AUC following 10 mM paraquat exposure. Pesticide-only groups represent the corresponding locomotor-impairment baselines, and dashed horizontal lines indicate the mean negative-control AUC. All phytotherapeutic treatment groups remained below the negative-control reference. Each botanical constituent was administered at 0.1% (w/v); therefore, total crude-extract concentration differed among monotherapy, binary, and ternary formulations.

*Mucuna pruriens*-based binary and ternary formulations were associated with higher locomotor AUC values than the corresponding pesticide-only baselines in both the 2.0% glyphosate and 10 mM paraquat models (Table 7; Figure 7). However, the magnitude of improvement varied among formulations and pesticide models. Following exposure to 2.0% glyphosate, the pesticide-only group had a mean AUC of 5.009 ± 2.369, corresponding to 42.5% of negative-control performance. Treatment with 0.1% *M. pruriens* increased mean AUC to 6.273 ± 0.440, corresponding to 53.3% of negative-control performance, a relative improvement of 25.2%, and 18.7% recovery toward normal. The binary formulation containing *M. pruriens* + *C. pepo* produced a mean AUC of 6.993 ± 0.550, corresponding to 59.4% of negative-control performance, 39.6% relative improvement, and 29.3% recovery toward normal. *Mucuna pruriens* + *A. hypogaea* produced a mean AUC of 7.385 ± 0.679, corresponding to 62.7% of negative-control performance, 47.4% relative improvement, and 35.1% recovery toward normal. The ternary *M. pruriens* + *C. pepo* + *A. hypogaea* formulation produced the highest numerical AUC in the glyphosate model at 7.780 ± 0.607, corresponding to 66.1% of negative-control performance, 55.3% relative improvement, and 40.9% recovery toward normal. Following 10 mM paraquat exposure, the pesticide-only group had a mean AUC of 5.472 ± 1.074, representing 46.5% of negative-control performance. Treatment with *M. pruriens* alone increased mean AUC to 6.378 ± 0.674, corresponding to 54.1% performance, 16.6% relative improvement, and 14.4% recovery toward normal. Among the paraquat combination groups, *M. pruriens* + *C. pepo* produced a mean AUC of 7.746 ± 0.410, corresponding to 65.8% of negative-control performance and 36.1% recovery toward normal. *Mucuna pruriens* + *A. hypogaea* produced the highest numerical AUC at 7.876 ± 0.439, corresponding to 66.9% performance and 38.1% recovery toward normal. The ternary formulation produced a mean AUC of 7.492 ± 0.584, corresponding to 63.6% performance and 32.0% recovery toward normal. The blocked analysis of the combination-treatment dataset showed a significant overall treatment effect (*p* < 0.001), whereas experimental phase was not significant (*p* = 0.387). Among the 2.0% glyphosate combination groups, the ternary formulation had a significantly higher AUC than *M. pruriens* + *C. pepo* after Tukey adjustment (adjusted *p* = 0.008). The *M. pruriens* + *A. hypogaea* group did not differ significantly from either of these formulations. No pairwise differences among the three paraquat combination formulations remained statistically significant after Tukey adjustment. Because each constituent was administered at 0.1%, total crude-extract concentration increased from 0.1% with *M. pruriens* monotherapy to 0.2% in binary formulations and 0.3% in the ternary formulation. Consequently, higher AUC values in combination groups cannot be attributed specifically to additive or synergistic pharmacological interactions. The findings should instead be interpreted as treatment-associated numerical differences among formulations of unequal total extract concentration.

**TABLE 7:** Vial-level locomotor outcomes following *Mucuna pruriens*-based formulations in pesticide-exposed *Drosophila melanogaster*.

Pesticide model	Treatment/formulation	Total crude extract (%)	Mean AUC ± SD	Performance relative to negative control (%)	Relative improvement (%)	Recovery toward normal (%)
Glyphosate 2.0%	Pesticide only	—	5.009 ± 2.369	42.5	—	—
Glyphosate 2.0%	MP	0.1	6.273 ± 0.440	53.3	25.2	18.7
Glyphosate 2.0%	MP + CP	0.2	6.993 ± 0.550	59.4	39.6	29.3
Glyphosate 2.0%	MP + AH	0.2	7.385 ± 0.679	62.7	47.4	35.1
Glyphosate 2.0%	MP + CP + AH	0.3	7.780 ± 0.607	66.1	55.3	40.9
Paraquat 10 mM	Pesticide only	—	5.472 ± 1.074	46.5	—	—
Paraquat 10 mM	MP	0.1	6.378 ± 0.674	54.1	16.6	14.4
Paraquat 10 mM	MP + CP	0.2	7.746 ± 0.410	65.8	41.6	36.1
Paraquat 10 mM	MP + AH	0.2	7.876 ± 0.439	66.9	43.9	38.1
Paraquat 10 mM	MP + CP + AH	0.3	7.492 ± 0.584	63.6	36.9	32.0

MP, mucuna pruriens; CP, cucurbita pepo; AH, *Arachis hypogaea*. Each botanical constituent was administered at 0.1% (w/v); consequently, total crude-extract concentration was 0.1% for MP, monotherapy, 0.2% for binary formulations, and 0.3% for the ternary formulation. Relative improvement and recovery toward normal were calculated relative to the corresponding pesticide-only baseline and negative-control AUC, respectively. Because total extract concentrations differed among formulations and CP, and AH, were not evaluated independently as monotherapies, numerical differences must not be interpreted as evidence of pharmacological synergy.

### 3.6 Objective assessment of recovery toward normal locomotor performance

To provide an objective assessment of whether treatment restored locomotor performance toward the normal reference, recovery across the ordered L1–L10 observations were evaluated relative to the negative-control trajectory. A treatment group was considered to have reached the normal range when its mean locomotor response was equal to or greater than the lower 95% confidence limit of the negative-control response at the corresponding L1–L10 observation. The first normal-range reading was defined as the earliest L1–L10 observation at which the treatment-group response reached or exceeded the lower 95% confidence limit of the corresponding negative-control response. The first sustained normal-range reading was defined as the earliest observation at which this criterion was achieved and subsequently maintained through all remaining observations up to L10. None of the *M. pruriens* monotherapy or combination-treatment groups reached the operational normal range at any L1–L10 observation. Consequently, neither a first normal-range reading nor a first sustained normal-range reading could be assigned for any treatment group (Table 8). These findings demonstrate that, although several phytotherapeutic treatments produced numerical improvement in vial-level AUC relative to their corresponding pesticide-only baselines, none restored locomotor performance to the predefined normal-reference range. The observed treatment effects should therefore be interpreted as partial locomotor improvement rather than complete functional recovery. The phytotherapeutic treatments produced partial improvement in locomotor performance relative to pesticide-only baselines; however, none of the monotherapy or combination groups reached the predefined normal-reference range across L1–L10, indicating incomplete functional recovery.

**TABLE 8:** Normal-range recovery assessment following phytotherapeutic treatment in pesticide-exposed *Drosophila melanogaster*.

Pesticide model/treatment set	Groups evaluated	First normal-range reading	First sustained normal-range reading	Recovery interpretation
Glyphosate monotherapy	0.5% glyphosate + MP; 1.0% glyphosate + MP; 2.0% glyphosate + MP	Not reached	Not reached	Partial improvement only
Glyphosate 2.0% combinations	MP + CP; MP + AH; MP + CP + AH	Not reached	Not reached	Partial improvement only
Paraquat 10 mM monotherapy	MP	Not reached	Not reached	Partial improvement only
Paraquat 10 mM combinations	MP + CP; MP + AH; MP + CP + AH	Not reached	Not reached	Partial improvement only

MP, mucuna pruriens; CP, cucurbita pepo; AH, *Arachis hypogaea*. The normal range was operationally defined as a mean locomotor response equal to or greater than the lower 95% confidence limit of the negative-control response at the corresponding L1–L10 observation. The first normal-range reading was the earliest observation meeting this criterion, whereas the first sustained normal-range reading required the criterion to remain satisfied through all subsequent observations. Because L1–L10 were ordered assay observations rather than confirmed clock-time measurements, these measures were not interpreted as recovery times.

## 4 Discussion

### 4.1 Principal findings

Crude *Mucuna pruriens* produced concentration-dependent mortality in adult *Drosophila melanogaster*, with 0.1% identified as the operational NOEL and 0.5% as the LOEL under seven-day exposure. Paraquat and glyphosate significantly impaired locomotor performance, whereas 0.1% *M. pruriens*, alone or in combination with *Cucurbita pepo* and *Arachis hypogaea*, produced partial locomotor improvement without restoration to the negative-control range. These findings support behavioural improvement rather than direct neuroprotection because dopaminergic, oxidative-stress, and neuronal endpoints were not measured.

### 4.2 Toxicity of *Mucuna pruriens*

Mortality increased from 13.9% in negative control to 45.6% at 2.0% *M. pruriens*, supporting concentration-dependent toxicity. Although 0.1% did not significantly increase mortality, this NOEL is specific to the present experimental conditions and should not be interpreted as a universal safe concentration. *Mucuna pruriens* contains L-DOPA, phenolics, flavonoids, alkaloids, and other bioactive constituents whose concentrations can vary substantially among preparations (Amro et al., 2018; Hammoud et al., 2025). Standardization and phytochemical characterization are therefore essential when interpreting both toxicity and potential therapeutic effects. The logistic and probit models estimated an LC_50_ of approximately 2.06%. However, mortality remained below 50% at the highest tested concentration of 2.0%; therefore, the estimate represents a model-dependent extrapolation rather than an experimentally observed threshold. Concentrations above 2.0% should be evaluated in future studies to better characterize the upper concentration–response curve.

### 4.3 Pesticide-associated locomotor impairment

The preliminary mortality experiment supported selection of 10 and 20 mM paraquat because these concentrations retained sufficient survival for behavioural assessment, whereas mortality exceeded 50% at 30 mM. Glyphosate concentrations of 0.5%–2.0% similarly preserved adequate survival. Paraquat- and glyphosate-associated locomotor dysfunction is consistent with experimental evidence linking pesticide exposure with oxidative stress, mitochondrial dysfunction, and behavioural impairment (Cattani et al., 2014; Costas-Ferreira et al., 2022; Dogan et al., 2025; Kishore et al., 2026; Quintero-Espinosa et al., 2024). The lower mean AUC observed at 10 mM than at 20 mM paraquat did not remain significant after multiplicity adjustment. Therefore, the findings do not demonstrate that 10 mM was more toxic than 20 mM and do not support an inverse or hormetic dose–response. Both concentrations caused substantial locomotor impairment, while the non-monotonic ordering may reflect biological and experimental variability. Importantly, the pesticide concentrations used here were experimental model-induction concentrations rather than human environmental exposure equivalents. Differences in exposure route, toxicokinetics, metabolism, body size, and exposure duration preclude direct translation to occupational or environmental human doses (Baltazar et al., 2014; da Silva et al., 2026; Tanner et al., 2011).

### 4.4 Locomotor effects of phytotherapeutic treatment

Treatment with 0.1% *M. pruriens* produced partial numerical improvement relative to pesticide-only baselines, but recovery toward normal remained limited and none of the treated groups reached the predefined normal range. Although *M. pruriens* is a natural source of L-DOPA and has been investigated experimentally and clinically for parkinsonian motor symptoms (Cilia et al., 2026; Cilia et al., 2017; Hammoud et al., 2025; Poddighe et al., 2014), the present study did not measure L-DOPA, dopamine, catecholamine metabolites, oxidative-stress markers, mitochondrial function, tyrosine hydroxylase, or dopaminergic-neuron integrity. Consequently, dopamine replacement, antioxidant activity, and neuronal protection remain plausible but unconfirmed mechanisms. Combination formulations also produced partial improvement. However, total crude-extract concentration increased from 0.1% with *M. pruriens* alone to 0.2% in binary combinations and 0.3% in the ternary formulation. Furthermore, *C. pepo* and *A. hypogaea* were not evaluated independently. Although both plants contain bioactive and antioxidant phytochemicals (Montesano et al., 2018; Peng et al., 2021; Toomer, 2018), the present design does not permit conclusions regarding pharmacological additivity or synergy.

### 4.5 Strengths, limitations, and future directions

Strengths of the study include prior dose-ranging toxicity assessment, empirical pesticide-dose selection, use of both endpoint mortality and Kaplan–Meier survival analysis, and vial-level AUC as the primary locomotor endpoint. Treating the vial as the experimental unit also reduced the risk of pseudoreplication. Several limitations remain. CMC at 0.5% was used in both pesticide and phytotherapeutic formulations, whereas the negative control received standard diet only; CMC-only control was not included. Methanol was removed during extraction, but residual solvent was not analytically quantified, and a methanol-only control was absent. Therefore, independent vehicles and residual-solvent effects cannot be excluded. The use of young adult males, non-standardized crude extracts, unequal total doses among formulations, and absence of *C. pepo*-only and *A. hypogaea*-only treatments further limit generalizability. Most importantly, the study provides behavioural rather than mechanistic evidence. Future studies should integrate negative geotaxis with ROS, malondialdehyde, glutathione, superoxide dismutase, catalase, dopamine and its metabolites, tyrosine hydroxylase, mitochondrial function, and direct assessment of dopaminergic neurons. Phytochemical standardization, L-DOPA quantification, vehicle and solvent controls, fixed-total-dose combination studies, and mammalian validation are also required before translational conclusions can be drawn. Current clinical evidence supports continued investigation of *M. pruriens*, but findings from standardized clinical preparations cannot be directly extrapolated to the crude extract evaluated here.

## 5 Conclusion

Crude *Mucuna pruriens* produced concentration-dependent toxicity in adult *Drosophila melanogaster*, with 0.1% identified as the operational NOEL and 0.5% as the LOEL under the seven-day exposure conditions. The model-derived LC_50_ of approximately 2.06% should be interpreted cautiously because it was extrapolated beyond the highest tested concentration. Paraquat and glyphosate significantly impaired locomotor performance, whereas treatment with 0.1% *M. pruriens*, alone or in combination with *C. pepo* and *A. hypogaea*, was associated with partial improvement relative to pesticide-only baselines. However, none of the treatments restored locomotor performance to the predefined normal range. The findings therefore provide evidence of treatment-associated behavioural improvement rather than direct neuronal or molecular neuroprotection. The apparent advantages of combination formulations should also be interpreted cautiously because total crude-extract concentrations differed among formulations and *C. pepo* and *A. hypogaea* were not evaluated independently. Future studies should incorporate vehicle and solvent controls, higher concentrations for LC_50_ determination, phytochemical standardization and L-DOPA quantification, fixed-total-dose combination designs, and biochemical, catecholaminergic, mitochondrial, and dopaminergic-neuron endpoints. These studies are necessary to clarify mechanisms, establish reproducibility and safety, and determine the translational relevance of *M. pruriens*-based formulations.

## Data Availability

The raw data supporting the conclusions of this article will be made available by the authors, without undue reservation.
